# Antiviral Effect of Budesonide against SARS-CoV-2

**DOI:** 10.3390/v13071411

**Published:** 2021-07-20

**Authors:** Natalie Heinen, Toni Luise Meister, Mara Klöhn, Eike Steinmann, Daniel Todt, Stephanie Pfaender

**Affiliations:** 1 Department of Molecular and Medical Virology, Ruhr-University Bochum, 44801 Bochum, Germany; Natalie.Heinen@rub.de (N.H.); Toni.Meister@rub.de (T.L.M.); Mara.Kloehn@rub.de (M.K.); Eike.Steinmann@rub.de (E.S.); Daniel.Todt@rub.de (D.T.); 2 European Virus Bioinformatics Center (EVBC), 07743 Jena, Germany

**Keywords:** SARS-CoV-2, antivirals, budesonide, variants of concern, corticosteroids

## Abstract

Treatment options for COVID-19, a disease caused by *Severe* *Acute* *Respiratory* *Syndrome* Coronavirus 2 (SARS-CoV-2) infection, are currently severely limited. Therefore, antiviral drugs that efficiently reduce SARS-CoV-2 replication or alleviate COVID-19 symptoms are urgently needed. Inhaled glucocorticoids are currently being discussed in the context of treatment for COVID-19, partly based on a previous study that reported reduced recovery times in cases of mild COVID-19 after inhalative administration of the glucocorticoid budesonide. Given various reports that describe the potential antiviral activity of glucocorticoids against respiratory viruses, we aimed to analyze a potential antiviral activity of budesonide against SARS-CoV-2 and circulating variants of concern (VOC) B.1.1.7 (alpha) and B.1.351 (beta). We demonstrate a dose-dependent inhibition of SARS-CoV-2 that was comparable between all viral variants tested while cell viability remains unaffected. Our results are encouraging as they could indicate a multimodal mode of action of budesonide against SARS-CoV-2 and COVID-19, which could contribute to an improved clinical performance.

## 1. Introduction

*Severe Acute Respiratory Syndrome* Coronavirus 2 (SARS-CoV-2) emerged in December 2019 in China [1] and continues to spread worldwide with a tremendous velocity that requires an unprecedented scientific and pharmaceutical effort to develop novel treatment options and to re-evaluate existing treatments to fight its associated disease, COVID-19. As of 21 April 2020, the National Institute of Health (NIH) recommends treatment with bamlanivimab plus etesevimab [2] or casirivimab plus imdevimab and the FDA-approved antiviral agent remdesivir [3] for outpatients with mild to moderate COVID-19 symptoms and for patients who require supplemental oxygen, respectively. However, due to uncertainty regarding the clinical benefits in severe patients who require high-flow oxygen, noninvasive or invasive mechanical ventilation, remdesivir is not routinely recommended for treatment in these patients [4,5]. Evidently, the development of effective drugs for treatment, especially for individuals suffering from severe symptoms of SARS-CoV-2 infection is becoming increasingly urgent. In addition, the worldwide emergence of novel variants of concern (VOC), including the variant B.1.1.7 (alpha) and variant B.1.351 (beta) [6,7] with enhanced transmission kinetics and/or altered immune recognitions further necessitates rapid and effective antiviral strategies that can complement current vaccination efforts in order to control the current pandemic.

A recent study by Ramakrishnan et al. reported reduced recovery times in cases of mild COVID-19, analyzed in a randomized phase 2 trial, after inhaling a total daily dose of 1600 µg budesonide [8]. Interestingly, this study was inspired by the observation that the prevalence of chronic respiratory diseases among patients with COVID-19 appeared to be lower compared to the general population [9]. Budesonide, a non-halogenated glucocorticoid, is a broadly used anti-inflammatory drug and is commonly applied via inhalation to treat respiratory diseases, such as adult and childhood asthma bronchiale [10,11], and moderate-to-severe chronic obstructive pulmonary disease (COPD) [12]. In addition to anti-inflammatory effects, budesonide has been shown to exert an in vitro antiviral activity against human rhinovirus infection by activation of autophagy via enrichment of reactive oxygen species [13] and has recently been demonstrated to reduce the cytopathic effect of *Middle East Respiratory Syndrome* (MERS)-CoV infected cells [14]. Along those lines, an earlier study reported an inhibitory effect of budesonide in combination with glycopyrronium and formoterol against the seasonal human coronavirus (HCoV)-229E [15].

However, a direct effect on SARS-CoV-2 has not yet been studied to the best of our knowledge. Hence, we examined a potential antiviral effect of pure compound budesonide and the budesonide suspension Pulmicort^®^ against the SARS-CoV-2 WT strain B1.1.70 and the circulating VOCs B.1.1.7 (alpha) and B1.351 (beta) in vitro. Limited dilution assay was employed to determine viral titers post treatment while cell viability was monitored by LDH release. Our results suggest that treatment with budesonide reduces titers of SARS-CoV-2 and VOCs significantly while cell viability remains unaffected.

## 2. Materials and Methods

Vero E6 cells were seeded at a density of 30,000 cells per well in a 24 well plate and were maintained at 37 °C and 5% CO_2_ overnight in DMEM containing 10% fetal calf serum (FCS), 1% L-Glutamine, 1% non-essential amino acids (NEAA) and 1% Penicillin/Streptomycin. Subsequently, cells were treated with budesonide (Selleck Chemicals; dissolved in DMSO, with amounts equivalent to 0.25 % of total volume) or Pulmicort^®^ (AstraZeneca; suspension containing 0.5 mg budesonide per 2 mL) in concentrations of 0.1, 1, 5 and 25 µM. Control cells were treated with equal amounts of solvent (0.25% DMSO). Simultaneously, cells were infected with the SARS-CoV-2 WT strain B1.1.70 (GISAID accession ID: EPI_ISL_1118929) or the circulating variants of concern B.1.1.7 (alpha, GISAID accession ID: EPI_ISL_751799), or B1.351 (beta, GISAID accession ID: EPI_ISL_803957) at a MOI of 0.1. Viral titers were determined by limited dilution assay as TCID_50_/mL and calculated by the Spearman–Kärber method 24 h post infection. Cytotoxicity was measured with CytoTox 96^®^ non-radioactive cytotoxicity assay (Promega).

Statistical data analysis was performed using GraphPad Prism v9.1 (GraphPad Software, San Diego, CA, USA). Differences in mean titers were tested by ANOVA followed by Dunnett’s corrected t-test versus respective controls. Four parameter log-logistic regression analyses were performed using the least squares fitting method.

## 3. Results

**Figure 1:**
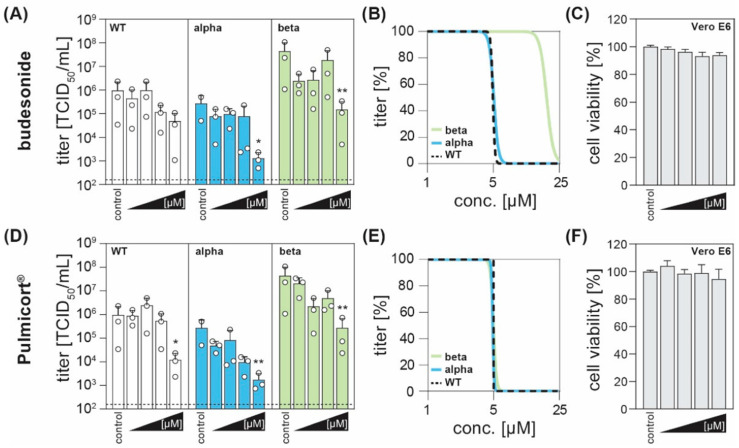
The corticosteroid budesonide acts antiviral against SARS-CoV-2 in vitro. (**A**,**D**) Vero E6 cells were infected with the SARS-CoV-2 variants B1.1.70 (WT, white), B.1.1.7 (alpha, blue), or B1.351 (beta, green) at a MOI of 0.1 and simultaneously treated with increasing concentrations of budesonide (Selleck Chemicals; dissolved in DMSO) or Pulmicort^®^ (AstraZeneca; suspension containing 0.5 mg budesonide per 2 mL), in concentrations of 0.1, 1, 5 and 25 µM. 24 h post infection, viral titers were determined by limited dilution assay and are depicted as TCID_50_/mL (three independent experiments indicated by open circle with bars depicting mean +SD; * *p* < 0.05, ** *p* < 0.01, ANOVA followed by Dunnett’s corrected t-test versus respective controls). (**B**,**E**) Four parameter log-logistic regression analyses were performed using the least squares fitting method implemented in GraphPad Prism v9.1. (**C**,**F**) Cytotoxicity was measured with the CytoTox 96^®^ non-radioactive cytotoxicity assay (Promega). Depicted are the normalized mean +SD of three independent experiments.

In order to evaluate a potential antiviral effect of budesonide, Vero E6 cells were infected with SARS-CoV-2 WT (B.1.1.70) or the currently circulating VOC B.1.1.7 (alpha) and B.1.351 (beta) and simultaneously treated with budesonide (dissolved in DMSO) or Pulmicort^®^ (suspension containing 0.5 mg budesonide per 2 mL) in concentrations of 0.1, 1, 5 and 25 µM. We observed that treatment with higher concentrations of budesonide, both administered as pure compound (Figure 1A) or as active compound of the licensed drug Pulmicort^®^ (Figure 1D), resulted in an antiviral effect and reduced viral titers compared to the respective control for all viral variants tested. Upon treatment with Pulmicort^®^, concentrations of 25 µM reduced viral titers significantly for all three variants tested. In accordance to these data, non-linear regression revealed comparable results among all virus variants, with half maximal inhibitory concentrations (IC_50_) ranging between 4.8 and 20 µM (Figure 1B,E). Of note, a higher initial titer of the variant B.1.351 resulted in a minor shift of the IC_50_ (Figure 1B). Importantly, a cytotoxicity assay revealed no effects on cell viability of the administered compounds compared to the administration of the same amount of solvent (Figure 1C,F). In conclusion, we hereby provide evidence that budesonide can significantly reduce SARS-CoV-2 titers in vitro.

## 4. Discussion

Treatment options for COVID-19 patients are currently very limited. Remdesivir, an FDA-emergency use approved drug is currently no longer recommended for use in acutely ill patients [16]. Inhaled corticosteroids, commonly administered for a variety of pulmonary diseases, have been discussed for use in COVID-19 therapy. Recent observations by Ramakrishnan et al. suggest that the inhaled corticosteroid budesonide reduces clinical recovery times and prevents progression and clinical deterioration during mild COVID-19 infection [8]. To test whether pure compound budesonide or the budesonide suspension Pulmicort^®^ altered viral loads and viral progeny production of SARS-CoV-2 and its variant of concern B.1.1.7 (alpha) and B.1.351 (beta), we treated Vero E6 with budesonide and determined viral titers by limited dilution while monitoring cell viability by LDH release. In contrast to Ramakrishnan et al. [8], who were unable to show a significant reduction of viral loads between budesonide treated and usual care groups in vivo, we observed significant reduction of viral titers for all viral variants in vitro when cells were treated with 25 µM budesonide. These results are in accordance with previous studies that demonstrated the suppression of SARS-CoV-2 and MERS-CoV RNA copy number by targeting the viral replication–transcription complex in differentiated human bronchial tracheal epithelial cells by the inhaled corticosteroid ciclesonide [14]. However, whether budesonide employs similar mechanisms to reduce viral loads warrants further evaluation.

Interestingly, Yamaya et al. did not show a decrease in viral titers of the human coronavirus (HCoV-) 229E upon treatment with 0.1 µM budesonide in primary human nasal (HNE) and tracheal (HTE) epithelial cell cultures [15], which is consistent with our presented results, showing a decrease in viral titers only in higher concentrations of budesonide. These findings might suggest, that delivering a lower dosage of budesonide (for instance due to incorrect inhalation and use of inhalation devices) might have an impact on the detection of viral loads in patients. Moreover, the complexity of human tissue and the immune system plays an important role in vivo, which might have an impact on the antiviral activity of compounds, such as budesonide. Further experiments using more authentic model systems like human airway epithelial cell cultures or lung organoids are necessary to gain further insights into the antiviral activity of budesonide.

Although studies have shown that corticosteroids reduce viral loads [13,14,15], others have suggested that corticosteroids do not affect [17] or even slow coronavirus clearance [18,19,20], which has raised some general concern about corticosteroid admission in COVID-19 patients. Nevertheless, some corticosteroids (e.g., dexamethasone) have been found to improve survival in hospitalized patients, especially in patients who require mechanical ventilation [21,22]. Therefore, corticosteroids have only been recommended as treatment for SARS-CoV-2 for these subsets of patients by the NIH [4], which at the same time advise physicians to closely monitor potentially emerging side effects, that may include hyperglycemia, secondary infections, psychiatric effects and/or avascular necrosis [23]. Overall, these observations reinforce the fact that further studies investigating putative adverse side effects of patients receiving budesonide during COVID-19 infection are required.

COVID-19 has been associated with acute respiratory distress syndrome (ARDS), especially in severely ill patients [24]. Interestingly, some studies have investigated corticosteroids in the context of ARDS [25,26]. Mohamed and Meguid found that budesonide improved lung mechanics and oxygenation when patients with ARDS were nebulized with budesonide. They also observed a significant reduction of inflammation markers TNF-α, IL-1β, and IL-6 [26]. In fact, corticosteroids such as budesonide have been recognized in numerous past studies to exhibit anti-inflammatory and immunomodulatory activities. For instance, budesonide has been found to inhibit early innate antiviral immune responses in vitro, suggesting that corticosteroids might restrict excessive inflammation [27]. Moreover, various clinical trials with asthma patients demonstrated beneficial effects of inhaled budesonide on airway inflammation and airway hyperresponsiveness [28,29,30]. Nevertheless, further investigations on putative effects of budesonide on ARDS and inflammation in the context of SARS-CoV-2 infections remains to be elucidated.

To put our findings into perspective, results need to be interpreted with caution as pharmacokinetics and pulmonary absorption of drugs are detrimental to treatment outcome in patients. In addition, performing experiments with more incremental dose-response analysis of the antiviral effect of budesonide can narrow down the IC_50_ range.

Furthermore, with Vero E6 cells an immortalized cell culture model was employed, therefore results in primary cell culture or in vivo could differ. Nevertheless, these results are encouraging and could be explored in future studies upon inclusion of mechanistical analysis and authentic cell culture systems. The authors distance themselves from discussions about recommendations regarding administration of budesonide or similar inhaled corticosteroids as standard of care as the evaluation of potential beneficial or adverse effects in the clinical context were not within the scope of this study.

## Data Availability

The data generated and analyzed in this study are included in the article.
